# DockNet: high-throughput protein–protein interface contact prediction

**DOI:** 10.1093/bioinformatics/btac797

**Published:** 2022-12-09

**Authors:** Nathan P Williams, Carlos H M Rodrigues, Jia Truong, David B Ascher, Jessica K Holien

**Affiliations:** STEM College, RMIT University, Melbourne, VIC, Australia; Computational Biology and Clinical Informatics, Baker Heart and Diabetes Institute, Melbourne, VIC, Australia; School of Chemistry and Molecular Biosciences, University of Queensland, Brisbane, QLD, Australia; STEM College, RMIT University, Melbourne, VIC, Australia; Computational Biology and Clinical Informatics, Baker Heart and Diabetes Institute, Melbourne, VIC, Australia; School of Chemistry and Molecular Biosciences, University of Queensland, Brisbane, QLD, Australia; STEM College, RMIT University, Melbourne, VIC, Australia

## Abstract

**Motivation:**

Over 300 000 protein–protein interaction (PPI) pairs have been identified in the human proteome and targeting these is fast becoming the next frontier in drug design. Predicting PPI sites, however, is a challenging task that traditionally requires computationally expensive and time-consuming docking simulations. A major weakness of modern protein docking algorithms is the inability to account for protein flexibility, which ultimately leads to relatively poor results.

**Results:**

Here, we propose DockNet, an efficient Siamese graph-based neural network method which predicts contact residues between two interacting proteins. Unlike other methods that only utilize a protein’s surface or treat the protein structure as a rigid body, DockNet incorporates the entire protein structure and places no limits on protein flexibility during an interaction. Predictions are modeled at the residue level, based on a diverse set of input node features including residue type, surface accessibility, residue depth, secondary structure, pharmacophore and torsional angles. DockNet is comparable to current state-of-the-art methods, achieving an area under the curve (AUC) value of up to 0.84 on an independent test set (DB5), can be applied to a variety of different protein structures and can be utilized in situations where accurate unbound protein structures cannot be obtained.

**Availability and implementation:**

DockNet is available at https://github.com/npwilliams09/docknet and an easy-to-use webserver at https://biosig.lab.uq.edu.au/docknet. All other data underlying this article are available in the article and in its online supplementary material.

**Supplementary information:**

Supplementary data are available at *Bioinformatics* online.

## 1 Introduction

Over 300 000 protein–protein interaction (PPI) pairs have been identified in the human proteome. Technological advances, such as yeast two-hybrid screening (Van Criekinge and Beyaert, 1999) and affinity purification coupled with mass spectrometry (De Las Rivas and Fontanillo, 2010), have facilitated more high-throughput wet-laboratory identification of PPIs. However, these approaches are expensive, time-consuming and do not provide structural insights into the interaction. Those PPIs that have been elucidated, along with biochemistry techniques such as alanine scanning, have allowed for a better understanding of the features of a PPI. We know that globular PPI sites usually contain ‘hotspot residues’, which contribute around 85% of the binding interaction energy (Grosdidier and Fernández-Recio, 2008; Jubb *et al.*, 2015), and PPIs tend to be more hydrophobic (Korn and Burnett, 1991; Young *et al.*, 1994), have complementary geometrical structure (Jones and Thornton, 1996) and have specific amino acid features (Yan *et al.*, 2008).

Generally, protein docking algorithms are good at utilizing the structural and physicochemical properties to predict PPIs. However, due to computational expense, most algorithms treat each protein as a rigid body and optimize the relative conformation of each protein to promote shape complementation and minimize intermolecular energies (Dominguez *et al.*, 2003; Lyskov and Gray, 2008). Recently, machine learning has been used for PPI prediction and consistently outperforms other methods when measured on standard benchmarks (Fout, 2017; Sanchez-Garcia *et al.*, 2019; Townshend *et al.*, 2019; Xie and Xu, 2021). Here, we propose DockNet, a new method using a unique neural network architecture to tackle several key issues highlighted in the literature. DockNet has a user-friendly web server, providing a valuable tool for researchers to efficiently model the 3D structure of PPIs without extensive computational expertise.

## 2 Materials and methods

DockNet was constructed utilizing two datasets [DIPS (Xenarios *et al.*, 2000) and PPI4DOCK (Yu and Guerois, 2016)] to avoid overfitting and maximize the potential impact of the model, and performance comparison was performed using the benchmark DB5 dataset (Vreven *et al.*, 2015). The structures were pre-processed to extract five types of features (i) amino acid type, (ii) amino acid exposure (depth, solvent accessibility and half sphere exposure), (iii) pharmacophores, (iv) secondary structure type and (v) torsional angles (phi and psi). A hyperparameter search was performed where N models were trained sequentially i.e. the next hyperparameter combination would be selected based on previous results to maximize the AUC score of the validation set. A model was designed that, when given a pair of protein features, could output a matrix where each cell indicated the probability of two residues being in contact during a PPI. The model was trained with binary cross-entropy loss, weighted to counter the class imbalance caused by sparse contacts. Two augmentations, swapping the inputs and flipping the sequence order, allowed for four possible orientations of each protein pair and assisted in regularizing the model. See the Supplementary Methods for full details of the model construction. DockNet is implemented as a freely available user-friendly web server. The server front end is developed using the Materialize framework version 1.0.0, while the back end is built with Flask (version 1.0.2). The web server is hosted on a Linux Server running Nginx.

## 3 Results

The best-performing model featured a base of 128 convolution filters, two residual graph convolution layers, a dropout rate of 0.2, four wave blocks and contained 579 073 parameters. We attempted numerous augmentations of the model (Supplementary Table S1); however, there was no increase in performance, suggesting that our Siamese architecture was robust enough to abstract the PPI features independent of the order in which the monomers are input. Each model version trained on the full dataset took approximately 40 h on a V100 GPU, with approximately 102 ms taken per prediction on the test set. Furthermore, we assessed the prediction times of our DockNet model on an Intel Core i7 CPU with 2.60 GHz for proteins with sizes ranging from 134 to 1208 amino acids long. Processing times varied from 6.5 to 42.3 s to load the model and making predictions, and 16.3 to 187.1 s when we included time for pre-processing structures for feature calculations.

**Fig. 1.**
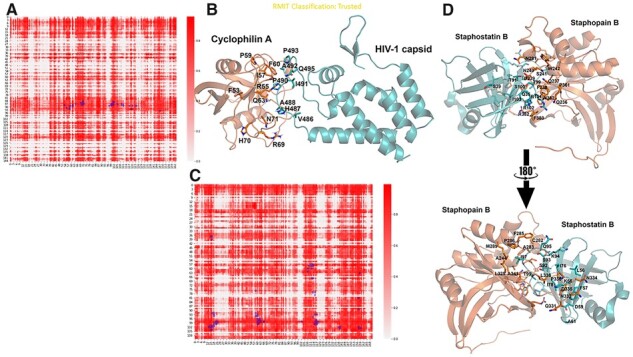
Performance of DockNet on rigid-body (**A** and **B**) and difficult (**C** and **D**) PPI from the DB5 database. (A and C) The predicted pairwise residue contact probability matrix for the interactions between HIV-1 capsid and cyclophilin A protein (A), Staphostatin B and Staphopain B (C). Structure of the complexes are shown in (B) (PDB: 1ak4) and (D) (PDB: 1pxv). Interface residues are highlighted as sticks on the protein structure and marked as squares on the heatmap

Comparison of our method to three other algorithms trained on the DIPS dataset, and therefore were direct comparisons to DockNet; Graph average (Fout, 2017), BIPSPI (Sanchez-Garcia *et al.*, 2019) and Siamese Atomic Surfacelet Network (Townshend *et al.*, 2019), showed DockNet achieves comparable results with a simpler architecture (Supplementary Table S2). DockNet was able to perform as well across all protein targets, with examples in each category (rigid body, medium and difficult) obtaining AUC scores over 0.85 (Supplementary Table S3). A rigid body [Cyclophilin A bound to HIV-1 capsid (Gamble *et al.*, 1996)] and difficult [*Staphylococcus aureus* Staphopain B bound to its inhibitor Staphostatin B (Filipek *et al.*, 2003)] examples are shown in Figure 1.

For the webserver, users upload a file in PDB format or provide a valid PDB accession code with the structure for two protein partners. The output page (Supplementary Fig. S2) summarizes the results for each protein partner on separate tabs, where predictions are averaged per residue and mapped onto the protein sequence using the FeatureViewer component (Garcia *et al.*, 2014) and the 3D structure via an interactive viewer built using NGLviewer (Rose *et al.*, 2018). In addition, a pairwise residue contact probability matrix is shown to help users to compare contact potentials between the input proteins. Finally, PDB structures for both partners with predicted probabilities for each residue annotated on the b-factor column are available for download, as well as the pairwise contact matrix as comma separated file (csv).

In summary, DockNet is an efficient neural network architecture which can predict contact residues between two interacting protein structures. DockNet captures the full context of the protein, leading to a prediction of interaction between 3D structures, rather than 2D graphs.

## Supplementary Material

btac797_Supplementary_Data

## References

[btac797-B1] De Las Rivas J. , Fontanillo C. (2010) Protein–protein interactions essentials: key concepts to building and analyzing interactome networks. PLoS Comput. Biol., 6, e1000807. 20589078 10.1371/journal.pcbi.1000807 PMC2891586

[btac797-B2] Dominguez C. et al (2003) HADDOCK: a protein−protein docking approach based on biochemical or biophysical information. J. Am. Chem. Soc., 125, 1731–1737. 12580598 10.1021/ja026939x

[btac797-B3] Filipek R. et al (2003) The staphostatin-staphopain complex: a forward binding inhibitor in complex with its target cysteine protease. J. Biol. Chem., 278, 40959–40966. 12874290 10.1074/jbc.M302926200

[btac797-B4] Fout A.M. (2017) Protein Interface Prediction Using Graph Convolutional Networks. In *NIPS'17: Proceedings of the 31st International Conference on Neural Information Processing Systems*. pp. 6533–6542.

[btac797-B5] Gamble T.R. et al (1996) Crystal structure of human cyclophilin a bound to the amino-terminal domain of HIV-1 capsid. Cell, 87, 1285–1294. 8980234 10.1016/s0092-8674(00)81823-1

[btac797-B6] Garcia L. et al (2014) J. FeatureViewer, a BioJS component for visualization of position-based annotations in protein sequences. F1000Res., 3, 47. 24741440 10.12688/f1000research.3-47.v1 PMC3983936

[btac797-B7] Grosdidier S. , Fernández-Recio J. (2008) Identification of hot-spot residues in protein-protein interactions by computational docking. BMC Bioinformatics, 9, 447. 18939967 10.1186/1471-2105-9-447 PMC2579439

[btac797-B8] Jones S. , Thornton J.M. (1996) Principles of protein-protein interactions. Proc. Natl. Acad. Sci. USA, 93, 13–20. 8552589 10.1073/pnas.93.1.13 PMC40170

[btac797-B9] Jubb H. et al (2015) Flexibility and small pockets at protein-protein interfaces: new insights into druggability. Prog. Biophys. Mol. Biol., 119, 2–9. 25662442 10.1016/j.pbiomolbio.2015.01.009 PMC4726663

[btac797-B10] Korn A.P. , Burnett R.M. (1991) Distribution and complementarity of hydropathy in mutisunit proteins. Proteins: Struct. Funct. Bioinformatics, 9, 37–55. 10.1002/prot.340090106 2017435

[btac797-B11] Lyskov S. , Gray J.J. (2008) The RosettaDock server for local protein-protein docking. Nucleic Acids Res., 36, W233–W238. 18442991 10.1093/nar/gkn216 PMC2447798

[btac797-B12] Rose A.S. et al (2018) NGL viewer: web-based molecular graphics for large complexes. Bioinformatics, 34, 3755–3758. 29850778 10.1093/bioinformatics/bty419 PMC6198858

[btac797-B13] Sanchez-Garcia R. et al (2019) BIPSPI: a method for the prediction of partner-specific protein–protein interfaces. Bioinformatics, 35, 470–477. 30020406 10.1093/bioinformatics/bty647 PMC6361243

[btac797-B14] Townshend R. et al (2019) End-to-end learning on 3d protein structure for interface prediction. Adv. neural inf. process. syst., **32**, 15642–15651.

[btac797-B15] Van Criekinge W. , Beyaert R. (1999) Yeast two-hybrid: state of the art. Biol. Proced. Online, 2, 1–38. 12734586 10.1251/bpo16 PMC140126

[btac797-B16] Vreven T. et al (2015) Updates to the integrated protein-protein interaction benchmarks: docking benchmark version 5 and affinity benchmark version 2. J. Mol. Biol., 427, 3031–3041. 26231283 10.1016/j.jmb.2015.07.016 PMC4677049

[btac797-B17] Xenarios I. et al (2000) DIP: the database of interacting proteins. Nucleic Acids Res., 28, 289–291. 10592249 10.1093/nar/28.1.289 PMC102387

[btac797-B18] Xie Z. , Xu J. (2021) Deep graph learning of inter-protein contacts. Bioinformatics, 38, 947–953. 10.1093/bioinformatics/btab761 PMC8796373 34755837

[btac797-B19] Yan C. et al (2008) Characterization of protein-protein interfaces. Protein J., 27, 59–70. 17851740 10.1007/s10930-007-9108-x PMC2566606

[btac797-B20] Young L. et al (1994) A role for surface hydrophobicity in protein‐protein recognition. Protein Sci., 3, 717–729. 8061602 10.1002/pro.5560030501 PMC2142720

[btac797-B21] Yu J. , Guerois R. (2016) PPI4DOCK: large scale assessment of the use of homology models in free docking over more than 1000 realistic targets. Bioinformatics, 32, 3760–3767. 27551106 10.1093/bioinformatics/btw533

