# Supplementary material for: DockNet: high-throughput protein–protein interface contact prediction
Source: Bioinformatics. 2022 Dec 9;39(1):btac797. doi: 10.1093/bioinformatics/btac797 (PMC9825772; doi:10.1093/bioinformatics/btac797)
Supplement: btac797_Supplementary_Data [file btac797_supplementary_data.docx]

Supplementary methods

## Datasets

We combined three separate datasets to avoid overfitting and maximise the potential impact of the model. 30,000 samples were from DIPS [1], which consists of crystallised proteins extracted from the Protein Data Bank, with identical models used for bound and unbound inputs. We also use the entirety of PPI4DOCK [2] which consists of homology models for the unbound structures and crystallised complexes for the bound target. DIPS and PPI4DOCK are only used for training the model. Finally, docking benchmark 5 (DB5) [3], which is composed of separate crystallised structures for both unbound and bound structures and is the current gold standard benchmark for this task. DB5 was split equally into a validation set, used to measure the performance of different hyperparameter combinations, and a test set which was used only for the final performance benchmark.

The unbound structures are pre-processed to extract 5 types of residue wise features that embed physical and chemical characteristics: (1) amino acid type, (2) residue exposure (depth, solvent accessibility and half sphere exposure), (3) pharmacophores, (4) secondary structure type and (5) torsional angles (phi and psi). A binary adjacency matrix is created by adding edges between residues that are within 6Å of each other in the unbound structure. For the label matrices, residues are determined to be in contact if they are within 8Å of each other in the bound crystallised complex.

To determine the optimal combination of model hyperparameters, a hyperparameter search was performed using a single V100 GPU. N models were trained sequentially, where the next hyperparameter combination would be selected by human supervision based on previous results to maximise the AUC score as evaluated on the validation set. The model was trained with binary cross entropy loss, weighted to counter the class imbalance caused by sparse contacts. Two augmentations, swapping the inputs and flipping the sequence order, allowed for four possible orientations of each protein pair and assisted in regularising the model.

## Neural Network Architecture

A model was designed that, when given a pair of protein features, could output a matrix where each cell indicated the probability of two residues being in contact during a protein-protein interaction. Each protein feature set consisted of a two-dimensional tensor of shape $\left| L \right|\times f,$ where $f$ represents the number of input features and $\left| L \right|$ is the number of residues in the protein, as well as an adjacency matrix of dimensions $\left| L \right|\times\left| L \right|$, to represent the protein’s 3D geometric structure. The output matrix $Y'$ is of dimensions $\left| L_{1} \right|\times\left| L_{2} \right|$, where ${Y'}_{l_{1}l_{2}}$ represents the probability of residues $l_{1}\in L_{1}$ and $l_{2}\in L_{2}$ being in contact.

We designed a backbone neural network architecture based on graph and two-dimensional convolutions and treated finer details of the network structure as hyperparameters. The network can be segmented into 3 distinct sections (Supplementary Figure 1). The first section consists of graph convolutional layers [4] with residual connections applied to each set of protein features individually. The number of layers and convolutional filters were treated as a hyperparameter. Secondly, after each set of features completes the forward pass through the graph convolution branch, the outputs are combined into a single three-dimensional tensor of shape $\left| L_{1} \right|\times\left| L_{2} \right|\times c$, where c is the number of convolutional filters used. The tensors are combined along the $\left| L \right|$ dimension index wise. Effectively, each residue’s feature set is individually aggregated with the feature set of each residue in the other protein. Two methods of aggregation are proposed, and the choice of method is treated as a hyperparameter. The first method concatenates the feature sets and passes them through a one-dimensional convolution layer to maintain consistent depth. The second effectively multiplies each index together by performing matrix multiplication on one-dimensional vector slices.

The third and final section of the network is fully composed of two-dimensional convolutional layers. We constructed an abstract composition of layers we label as a wave block. Using a 1x1 convolution of depth c, a dilated 3x3 convolution of depth $c/2$, another 1x1 convolution of depth c and a residual connection, this pattern is repeated 4 times with dilation rates of 1,2,4 and 8 respectively to compose a single wave block. The dilated structure is designed to capture long range interactions in the sequences within the hardware limitations. The number of wave blocks is treated as a hyperparameter. The final layer features a 1x1 convolution followed by a sigmoid activation layer to output a probability $p_{ij}\in[0,1]$ representing the probability of contact between residues $i$ and $j$. Throughout the entire network, each graph convolution or two-dimensional convolutional layer is followed by an instance normalisation layer and a swish activation layer in respective order.

## Evaluation Metrics

Performance assessment for all evaluation metrics follows the rationale described in previous studies (e.g. [5]). Each metric is calculated separately per protein-protein complex and final results are reported as median values. This approach intends to prevent results for large complexes from having a disproportionate effect on the overall performance.


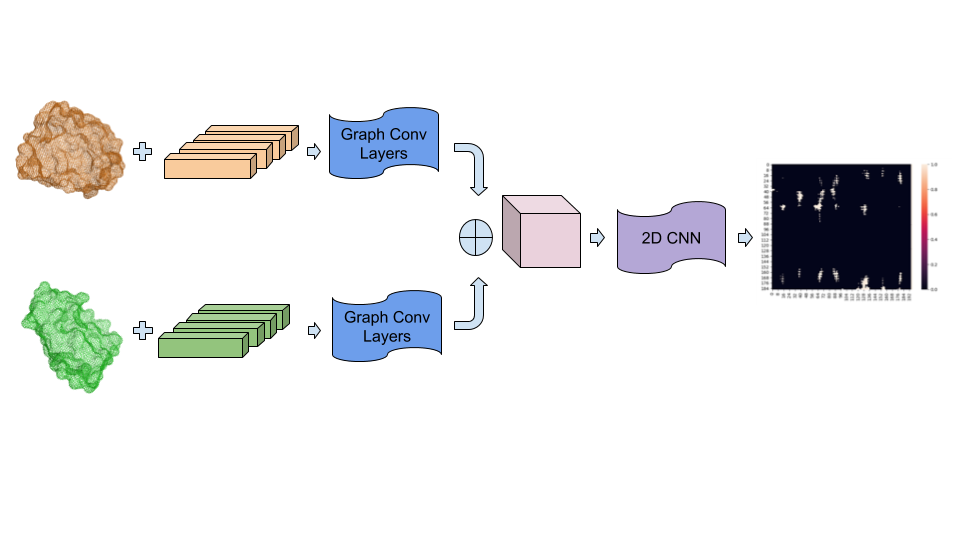


**Supplementary Figure 1: DockNet architecture**. DockNet’s workflow can be summarised into 3 main parts: (1) the structure of each monomer as well as protein features are fed into a graph convolution layer separately; (2) outputs are then combined into a single tensor followed by (3) a two-dimension convolution layer (wave block) for capturing long range interactions between protein sequences. The network’s output comprises a 2x2 matrix where each cell represents the probability of two residues from the two input proteins being in contact.


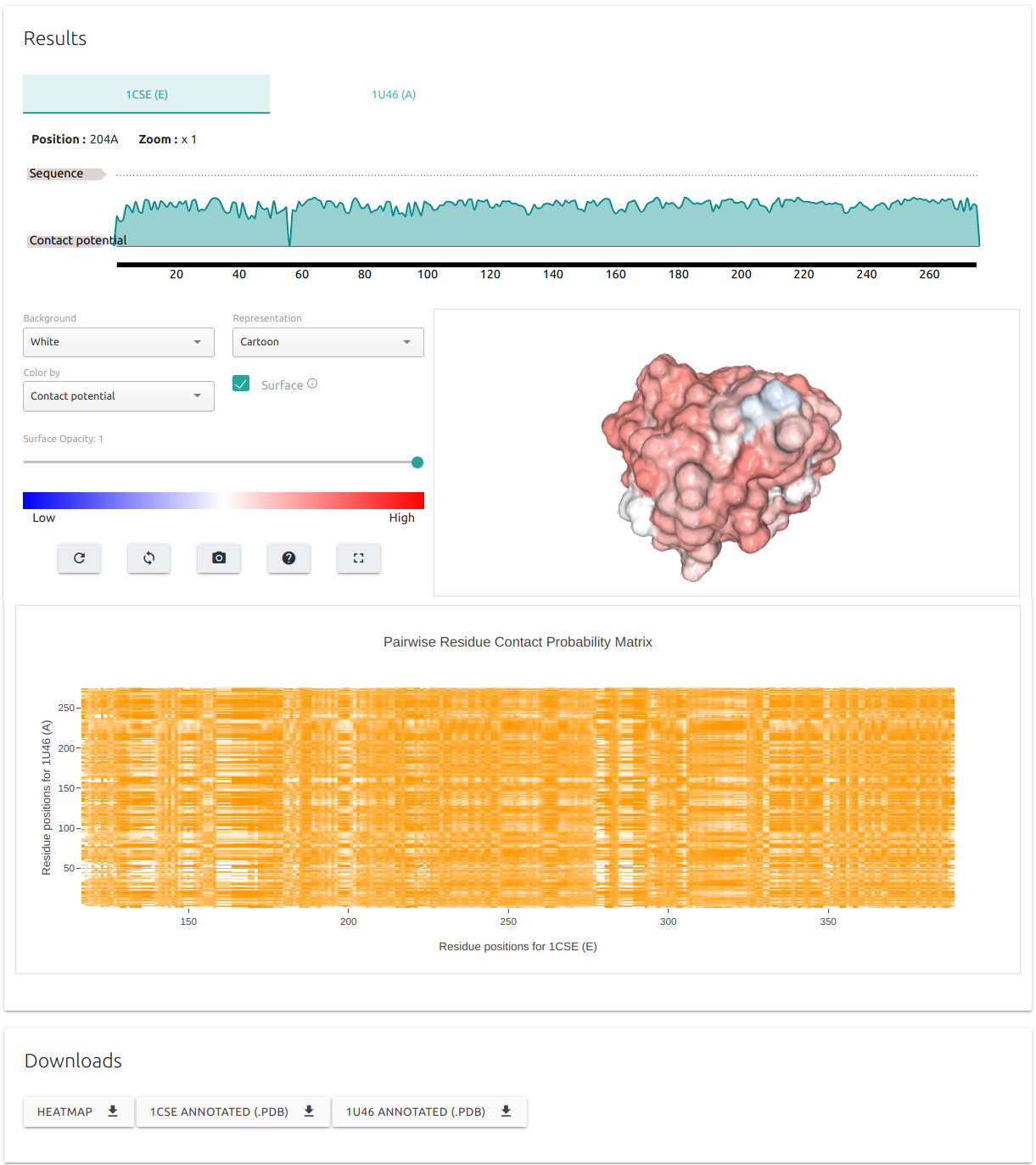


**Supplementary Figure 2: Results page for the DockNet webserver.** Probability of interaction is summarised per residues by averaging all probabilities for a single position. These are then mapped on to the protein sequence and also to the input 3D structures as an interactive viewer. Users may change visibility for specific partners using the tabs on the top of the page. Results are also described as a pairwise residue contact probability matrix, which gives the probability of two

Supplementary Table 1: Summary of different augmentation experiments

| Augmentation | AUC | RMSE | TPR | FPR |
| --- | --- | --- | --- | --- |
| Swap inputs | 0.84 | 0.44 | 0.89 | 0.95 |
| Flip first input | 0.81 | 0.42 | 0.86 | 0.92 |
| Flip second input | 0.81 | 0.43 | 0.87 | 0.93 |
| Swap & Flip first input | 0.80 | 0.42 | 0.85 | 0.91 |
| Swap & Flip second input | 0.80 | 0.41 | 0.85 | 0.92 |
| Flip both inputs | 0.77 | 0.42 | 0.81 | 0.88 |
| Swap & flip both inputs | 0.77 | 0.42 | 0.81 | 0.88 |
| All Augmentations | 0.77 | 0.39 | 0.83 | 0.87 |

Supplementary Table 2: Performance comparison of DockNet versus alternative methods. Results are shown in terms of median per-complex AUC on the test set.

| Methods | AUC |
| --- | --- |
| DockNet | 0.845 |
| SASNet | 0.982 |
| BIPSPI | 0.836 |
| Graph average | 0.712 |

**Supplementary Table 3: Examples protein-protein interaction prediction partners.** Categories are described in [3] Enzyme-Inhibitor (EI), Enzyme-Substrate (ES), Enzyme complex with regulatory or accessary chain (ER), Antibody-Antigen (AA), Antibody-Single domain Antibody (AS), G-protein containing others (OG), Receptor containing others (OR), miscellaneous others (OX).

| PPI | Difficulty level | Category | Partner 1 | Partner 2 | I-RMSD (Å) | AUC |
| --- | --- | --- | --- | --- | --- | --- |
| 2sni (E:I) | Rigid body | EI | 1ubn (A) | 2ci2 (I) | 0.35 | 0.93 |
| 1ak4 (A:D) | Rigid body | OX | 2cpl | 4j93 (A) | 0.87 | 0.92 |
| 2sic (E:I) | Rigid body | EI | 1sup | 3ssi | 0.36 | 0.90 |
| 3pc8 (A:C) | Rigid body | ER | 3pc6 (A) | 3pc7 (A) | 0.5 | 0.89 |
| 7cei (A:B) | Rigid body | EI | 1unk (D) | 1m08 (B) | 0.7 | 0.89 |
| 1r0r (E:I) | Rigid body | EI | 1scn (E) | 2gkr (I) | 0.45 | 0.86 |
| 1vfb (AB:C) | Rigid body | AA | 1vfa (AB) | 8lyz | 1.02 | 0.85 |
| 2hle (A:B) | Rigid body | OR | 2bba (A) | 1iko (P) | 1.4 | 0.84 |
| 1j2j (A:B) | Rigid body | OG | 1o3y (A) | 1oxz (A) | 0.63 | 0.84 |
| 1us7 (A:B) | Rigid body | ER | 2fxs (A) | 2w0g (A) | 1.06 | 0.84 |
| 1hcf (AB:X) | Rigid body | OR | 1b98 (AM) | 1wwb (X) | 0.88 | 0.82 |
| 4hx3 (BD:A) | Rigid body | EI | 4hwx (AB) | 1c7k (A) | 0.9 | 0.81 |
| 1ppe (E:I) | Rigid body | EI | 1btp | 1lu0 (A) | 0.44 | 0.79 |
| 1ewy (A:C) | Rigid body | ES | 1gjr (A) | 1czp (A) | 0.8 | 0.78 |
| 1he1 (C:A) | Rigid body | OG | 1mh1 | 1he9 (A) | 0.93 | 0.79 |
| 2ayo (A:B) | Rigid body | ER | 2ayn (A) | 2fcn (A) | 1.39 | 0.78 |
| 1zhi (A:B) | Rigid body | OX | 1m4z (A) | 1z1 a (A) | 0.68 | 0.77 |
| 1fle (E:i) | Rigid body | EI | 9est (A) | 2rel (A) | 1.02 | 0.76 |
| 1s1q (A:B) | Rigid body | OX | 2f0r (A) | 1yj1 (A) | 0.98 | 0.76 |
| 1clv (A:I) | Rigid body | EI | 1jae (A) | 1qfd (A) | 0.86 | 0.75 |
| 2abz (B:E) | Rigid body | EI | 3i1u (A) | 1zfi (A) | 0.9 | 0.74 |
| 2j0t (A:D) | Rigid body | EI | 966c (A) | 1d2b (A) | 1.23 | 0.74 |
| 4cpa (A:I) | Rigid body | EI | 8cpa (A) | 1h20 (A) | 0.62 | 0.73 |
| 1buh (A:B) | Rigid body | EI | 1hcl | 1dks (A) | 0.75 | 0.73 |
| 1gl1 (A:I) | Rigid body | EI | 1k2i (1) | 1pmc (A) | 1.21 | 0.73 |
| 3h2v (A:E) | Rigid body | OX | 3myi (A) | 1wi6 (A) | 0.8 | 0.73 |
| 1kac (A:B) | Rigid body | OR | 1nob (F) | 1f5w (B) | 0.95 | 0.68 |
| 1xu1 (ABD:T) | Rigid body | OR | 1u5y (ABD) | 1xut (A) | 1.3 | 0.69 |
| 2uuy (A:B) | Rigid body | EI | 1hj9 (A) | 2uux (A) | 0.43 | 0.67 |
| 1gcq (B:C) | Rigid body | OX | 1gri (B) | 1gcp (B) | 0.92 | 0.59 |
| 2cfh (A:C) | Medium difficulty | OX | 1sz7 (A) | 2bjn (A) | 1.55 | 0.87 |
| 3s9d (B:A) | Medium difficulty | OR | 1n6u (A) | 1itf (A) | 1.69 | 0.76 |
| 1nw9 (B:A) | Medium difficulty | ER | 1jxq (A) | 2opy (A) | 1.97 | 0.72 |
| 3f1p | Difficult | OX | 1p97 (A) | 1x0o (A) | 2.52 | 0.91 |
| 1r8s | Difficult | OG | 1hur (A) | 1r8m (E) | 3.73 | 0.84 |
| 1pxv | Difficult | EI | 1x9y (A) | 1nyc (A) | 2.63 | 0.83 |
| 3fn1 | Difficult | ER | 2edi (A) | 2lq7 (A) | 3.65 | 0.74 |

1. Xenarios, I., et al., *DIP: the database of interacting proteins.* Nucleic Acids Res, 2000. **28**(1): p. 289-91.

2. Yu, J. and R. Guerois, *PPI4DOCK: large scale assessment of the use of homology models in free docking over more than 1000 realistic targets.* Bioinformatics, 2016. **32**(24): p. 3760-3767.

3. Vreven, T., et al., *Updates to the Integrated Protein-Protein Interaction Benchmarks: Docking Benchmark Version 5 and Affinity Benchmark Version 2.* J Mol Biol, 2015. **427**(19): p. 3031-41.

4. Kipf, T.N. and M. Welling, *Semi-supervised classification with graph convolutional networks.* arXiv preprint arXiv:1609.02907, 2016.

5. Townshend, R.J.L., et al., *End-to-End Learning on 3D Protein Structure for Interface Prediction*, in *33rd Conference on Neural Information Processing Systems (NeurIPS 2019)*. 2018: Vancouver, Canada.
